# Deep Learning Kinetic Modeling of the Catalytic Decomposition of Ammonia in Green Hydrogen Production: Effects of Catalyst Composition and Operating Variables

**DOI:** 10.1021/acsomega.5c11092

**Published:** 2026-04-10

**Authors:** Felipe Vicent Dalcamim, Isabela Belapetravicius, Fabio M. Cavalcanti, Reinaldo Giudici

**Affiliations:** † 67420 Universidade de São Paulo, Escola Politécnica, Department of Chemical Engineering, Av. Prof. Luciano Gualberto 380, São Paulo, SP 05508-010, Brazil; ‡ Department of Chemical Engineering, Center of Technology and Geosciences, Institute for Petroleum and Energy Research (LITPEG), Laboratory of Refining and Cleaner Technology (LabRefino/Lateclim), Federal University of Pernambuco, Recife, PE 50740-540, Brazil

## Abstract

The increasing demand for sustainable energy has accelerated the development of green hydrogen production technologies. Among these, the catalytic decomposition of ammonia stands out because of its efficient storage and transportation as well as its compatibility with existing infrastructure. Nevertheless, challenges in enhancing the reaction performance still hinder its large-scale implementation. To address these limitations and optimize the process, this work presents the development of a deep-learning-based artificial neural network to model ammonia conversion as a function of operating conditions and catalyst composition encoded directly into the network. The final model designed significantly outperformed traditional machine learning techniques and the smaller architectures tested. Deep learning was fundamental for achieving the lowest predictive errors (RMSE = 10.06 ppm and MAE = 7.98 ppm) and minimizing the difference between training and validation errors, indicating a high degree of stability and generalization. A comprehensive sensitivity analysis was also conducted and aligned with literature findings, revealing the model’s capacity to capture complex physicochemical patterns. Finally, validation on external data further confirmed its generalization capabilities. To the best of our knowledge, this is the first study to implement deep neural networks for modeling the catalytic decomposition of ammonia, including catalyst compositional features, while also contributing to the broader, still-emerging application of deep learning in catalytic systems.

## Graphical abstract

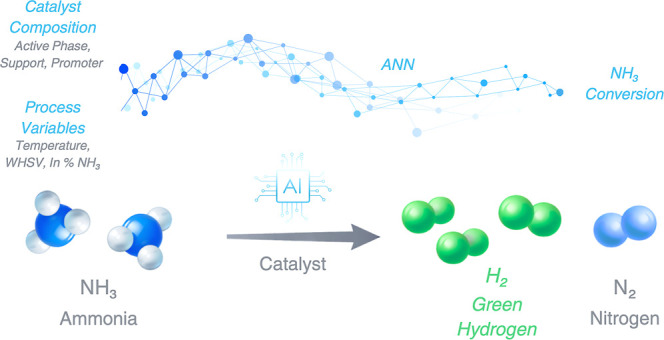

## 1 Introduction

The global energy matrix remains heavily dependent on fossil fuels, such as coal, oil, and natural gas, which account for approximately 84% of the global supply and contribute to over 65% of worldwide electricity production.^1^ Despite their economic convenience, these sources are major contributors to carbon emissions, intensifying climate change and its consequences.^2,3^ Reliance on fossil fuels also introduces geopolitical stress and economic vulnerabilities globally,^4^ particularly in energy-importing countries that face supply instability and price volatility.^5^ In response to these challenges, renewable and sustainable alternatives are now urgently needed and have become the focus of extensive research.^6,7^

Among the alternatives, hydrogen is particularly promising due to its capacity to support long-term energy storage, addressing intermittency issues associated with solar and wind energy.^8^ Hydrogen production is also significantly more space-efficient than extensive solar and wind farms. Currently, industrial hydrogen production is predominantly based on steam methane reforming, a cost-effective yet environmentally damaging process.^9^ In contrast, water electrolysis enables emission-free hydrogen generation but is constrained by high costs and low energy efficiency.^9^ In light of these limitations, the catalytic decomposition of ammonia, shown in eq 1, emerges as a compelling option. Ammonia is not only hydrogen-rich but also benefits from well-established storage, transportation, and safety practices and regulations.^10^

1 $$2{NH}_{3\left(g\right)}\to N_{2\left(g\right)}+3H_{2\left(g\right)}$$

However, the large-scale application of this process still faces technical challenges, as achieving high conversion rates requires elevated temperatures or ultraefficient catalysts, driving significant interest in its optimization.^11^ Traditional kinetic models have long been employed to describe chemical reactions, but their reliance on phenomenological assumptions and conservation laws often restricts their applicability to idealized conditions.^12^ In catalytic systems, these models tend to oversimplify the underlying mechanisms, failing to account for intricate details, such as reaction dynamics and the catalyst composition. Moreover, the implementation of more sophisticated models present in advanced simulation software is also limited by the considerable computational resources they require.

In this regard, Artificial Intelligence (AI) and Machine Learning (ML) technologies—particularly Artificial Neural Networks (ANNs)—demonstrate remarkable potential. Inspired by the human brain, ANNs are composed of interconnected neurons that adaptively learn from data, identifying the system nonlinearities and correlations without mechanistic equations.^13^ Unlike traditional theory-driven modeling, ANNs operate independently of explicit formulations of the laws governing the system: as data-driven models, they can describe catalytic reactions with a high degree of accuracy, even when the kinetic parameters or reaction pathways are partially unknown or involve complex dependencies among variables.^12,13^ Over the past two decades, ANNs have been increasingly and successfully employed in the modeling of chemical reaction systems, with studies validating their effectiveness in optimizing processes, selecting catalysts, and enhancing the overall reaction overall efficiency.

The pioneering work developed by Serra et al.,^13^ for instance, first highlighted the potential of ANNs in catalysis, predicting *n*-octane conversion and product yields during isomerization with impressive accuracy. Since then, other studies have extended ANNs to various applications, including the water–gas shift reaction,^14^ carbon monoxide oxidation,^15^ and methanol steam reforming.^16^ The models developed by these examples faithfully represented their respective systems, reducing the reliance on time-extensive experimental trials and enabling high-throughput screening of catalyst compositions and reaction conditions, paving the way for more efficient reaction and catalyst design.

Recent studies have also explored traditional ML techniques for modeling ammonia decomposition. Guo et al.^17^ applied Gradient Boosting to predict ammonia conversion, achieving strong predictive performance (RMSE = 13.24, MAE = 10.31, *R* ^2^ = 0.85) on a large data set of 1491 data points. The hydrogen formation rate was also modeled as an additional output, using a smaller data set containing 291 data points. Their model relied primarily on attributes or descriptors of catalyst properties, such as metal loading, average crystallite size, crystallinity index, specific surface area, pore volume, and average pore diameter, as well as operational conditions, namely, gas hourly space velocity (GHSV) and temperature. While the results already demonstrate the capacity of data-driven modeling, they reflect a focus on traditional ML methodologies and a limited description of catalyst composition—particularly given that several descriptors are inherently difficult to determine and catalysts with different compositions may present similar descriptor values despite displaying distinct performances—which is essential for catalyst design strategies.

In a distinct and, in many ways, complementary approach, this work explores deep learning strategies using an ANN model designed to analyze ammonia decomposition. Beyond textural descriptors, this study incorporates identities and relative contributions of all catalyst components—active phase, support, and promoter—encoded directly into the network. In addition to compositional features, process conditions such as the Weight Hourly Space Velocity (WHSV), ammonia feed concentration, and reaction temperature are also characterized. This broader scope enables the model to identify intrinsic catalyst composition-driven effects, while sensitivity analysis reveals the impact of subtle changes in the catalyst composition on the overall reaction efficiency, both of which are more directly aligned with catalyst formulation frameworks. To the best of our knowledge, this is the first study to implement an ANN-based model specifically designed for the catalytic decomposition of ammonia, considering the intrinsic chemical composition of the catalyst, rather than its textural descriptors, and the first study to explore deep learning architectures tailored to address the modeling of catalytic systems commonly characterized by highly diverse and limited data.

The following sections describe the collection, processing, and encoding of an experimental database extracted from the literature; the design and optimization of the model; the evaluation of its predictive accuracy; and finally, the analysis of correlations between process variables and their impact on reaction outcomes.

## 2 Materials and Methods

### 2.1 Data Collection, Processing, and Encoding

#### 2.1.1 Data Collection

The data set used in this work was derived from the literature review conducted by Lucentini et al. in 2019,^8^ which compiled 674 experiments on the catalytic decomposition of ammonia. The original database includes detailed descriptions of the main operating conditions: catalyst composition, including active phase, its weight fraction (Wt %), support, and promoter; feed flow parameters relative to the catalyst mass and reactor volume, represented by WHSV and GHSV, respectively; the ammonia concentration in the inlet stream (In % NH_3_), which in some experiments also contained inert gases; as well as the operating temperature. All experiments in the data set were performed at 1 bar. Furthermore, the database also provides the following process results: ammonia conversion (Conv. NH_3_), hydrogen production rate (H_2_ rate), apparent activation energy (*E*_a_), and turnover frequency (TOF).

Table S1 illustrates the original structure of part of the database, as presented in,^8^ highlighting the diversity in available information and the presence of missing data across various parameters.

#### 2.1.2 Data Processing

To address the issue of missing data, a processing phase was initiated, excluding variables that were insufficiently represented in the data set. Specifically, the GHSV, hydrogen production rate, apparent activation energy, and TOF were excluded because over 60% of the experiments lacked information for these parameters, rendering any attempt at extrapolation statistically unreliable. The high absence rates for these variables are likely due to the complexity and cost of their measurement. Following this step, experiments without specified ammonia conversion values were excluded, given that extrapolation, based on averages or inference from similar conditions, would directly introduce deviations from the actual reaction behavior. Additionally, the few experiments missing critical operating conditions, such as ammonia concentration in the feed stream (In % NH_3_) or WHSV, were also discarded due to their impact on reaction kinetics and catalyst performance.

At this stage, the catalyst composition remained predominantly qualitative, specifying only the presence of elements, oxides, or compounds in the active phase, support, and promoter, while weight fractions (wt %) were defined only for the active phase. In order to account for the exact catalyst composition, an extensive literature review was conducted, covering more than 90 articles from the original references cited in^8^ and retrieving missing weight fraction data for the support, promoter, and, in rare cases, the active phase. These values were often reported indirectly, typically as molar fractions or element ratios, demanding standardization.

When the information was not explicitly available, the support weight fraction was assumed to complement the total composition. This assumption is justified by the structural role of the support, which typically constitutes the largest fraction in heterogeneous catalysts.^8^ For cases where the active phase or promoter weight fractions were absent in the original study—which accounted for approximately 4% of the data set—extrapolation methods were applied, leveraging mode values from experiments within the same study or from studies with similar experimental conditions. Extrapolation was possible in these cases because of the substantial amount of data available for a robust statistical approach.

The entire processing phase resulted in the exclusion of 174 in some sense incomplete experiments from the original 674, resulting in a final 500-point large data set structured with the complete catalyst composition, including active phase, support, and promoter with their respective weight fractions; WHSV; inlet ammonia concentration (In % NH_3_); temperature; and ammonia conversion (Conv. NH_3_). Table S2, in correlation with Table S1, displays the same data points after processing.

#### 2.1.3 Data Encoding

Following the data processing phase, the categorical variables—active phase, support, and promoter—were numerically encoded to meet ANN input requirements. Both the active phase and the promoter were represented in elemental terms as their catalytic activity in the reaction is inherently linked to their metallic nature.^8^ In contrast, the support was encoded according to its original form (e.g., oxides and carbon-based structures) as its structural properties are crucial for the catalytic performance. For instance, carbon nanotubes (CNTs) and carbon nanofibers (CNFs) were maintained as distinct categories to preserve their unique morphological characteristics, which would be lost if they were simply represented as elemental carbon (C).

In the later stages of this study, the low statistical representation of certain variables in the original database was identified as a critical challenge. This limitation was observed in two specific contexts: catalyst compositional features with scarce representation and extreme operating conditions, typically characterized by unusually high or low temperatures and WHSV values rarely observed in the data set. Early validation results indicated that many experiments falling within these categories exhibited an exceptionally high prediction deviation. This behavior was attributed to the model’s limited ability to generalize in sparsely populated regions of the feature space. To address this challenge, several underrepresented catalyst formulations were decomposed into their constituent oxides whenever they were chemically meaningful. For example, sepiolite, originally treated as a single category, was encoded through its oxide components (SiO_2_, MgO, and Al_2_O_3_). This strategy increased the frequency of key descriptors and improved the model stability. In addition, experiments conducted under extreme or rarely represented operating conditions of temperature and WHSV were excluded from the final modeling data set. This restriction ensured that the model was developed within a statistically reliable domain, reducing the risk of overinterpreting trends driven by undersampling.

The final data set, after all processing and encoding steps, comprised 324 experiments. In this regard, it is important to note that despite the exclusions, the database remained highly diverse, encompassing more than 20 distinct metals for the active phase and the promoter, as well as 27 different support compounds, ensuring broad coverage of experimental conditions. A summary of the data is shown in Table 1.

**Table 1:** Summary of the Processed Database of Catalytic Decomposition of Ammonia with Operating and Compositional Variables, along with Their Range or Identity

variable	unit	range/identity
WHSV	mL g^–1^h^–1^	60–360,000
in % NH_3_	vol %	0.2–100
temperature	°C	300–650
conv. NH_3_	–	0–100
active phase^a^	wt %	Ba, Ca, Co, Fe, K, Li, Mg, Mn, Mo, N, Na, Ni, Ru, Sr, W, Zr
support^a^	wt %	AC, Al_2_O_3_, BaO, CMK3, CNFs, CNTs, CaO, C, CeO_2_, Fe_2_O_3_, GNP, K_2_O, La_2_O_3_, MCM41, MIL101, MgO, MnO_2_, Na_2_O, Nb_2_O_5_, OMC, Pr_6_O_11_, SBA15, SiO_2_, SrO, TiO_2_, Y_2_O_3_, ZrO_2_
promoter^a^	wt %	Al, Ba, Ca, Ce, Cs, K, La, Li, Mg, Na, Sr, Zr

a Categorical-Quantitative variables.

Table S3 illustrates a segment of the data set after encoding, which, aligned with Tables S1 and S2, showcases the same data points through the processing and encoding procedures.

### 2.2 Model Design

Fundamentally, the model was structured as a fully connected, feedforward neural network configured to predict ammonia conversion based on process and catalyst variables. Accordingly, the network inputs were assigned to a total of 50 input neurons: 3 corresponding to the operating conditions—WHSV, In % NH_3_, and temperature—and 47 corresponding to the encoded categories of the catalyst composition, which were represented by numerical arrays distributed across multiple neurons, as detailed previously. The output layer consisted of a single neuron corresponding to predicted ammonia conversion. Figure 1 presents a conceptual illustration of the ANN structure.

**Figure 1:**
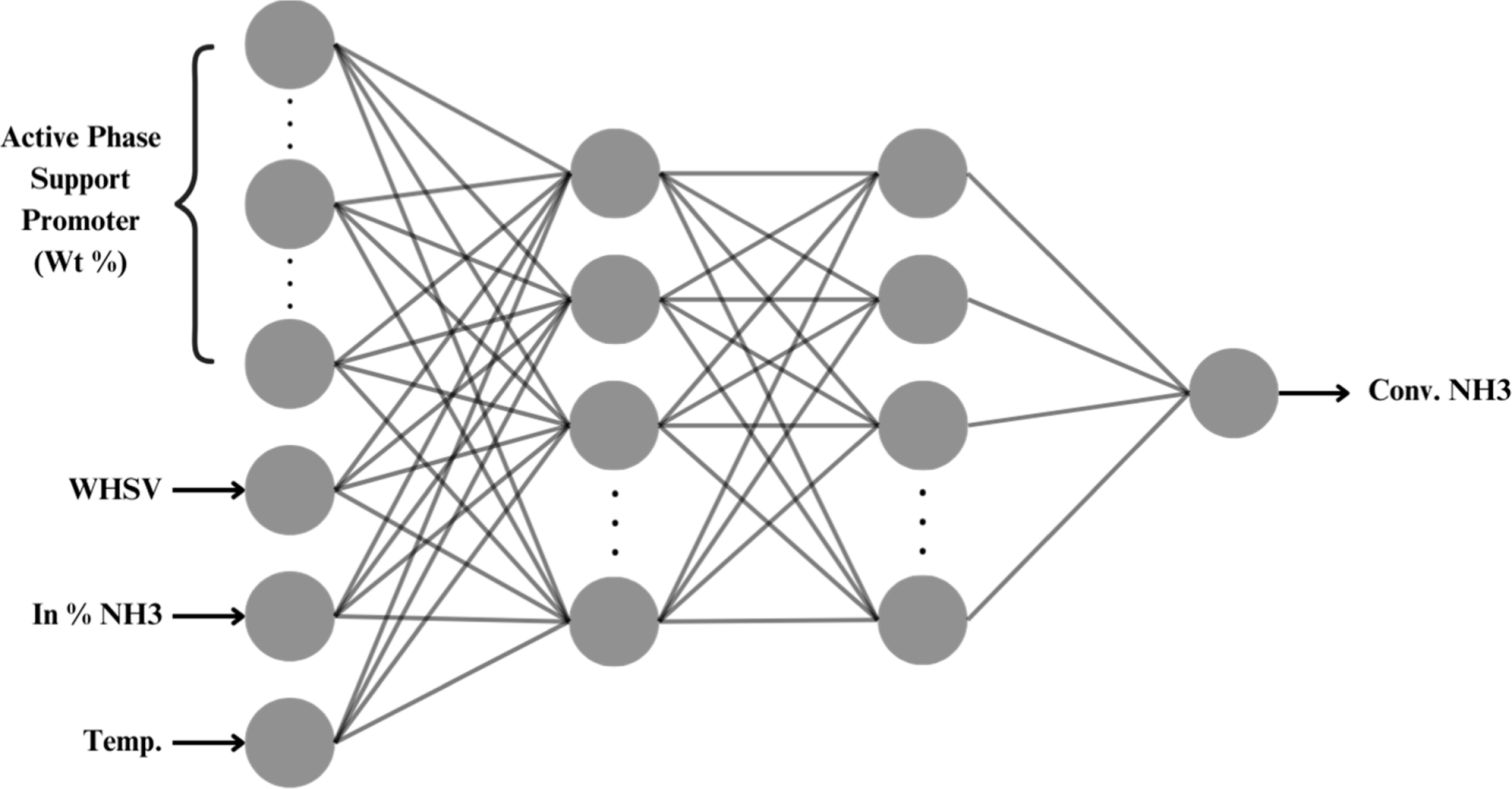
Conceptual structure of the network for analyzing the ammonia decomposition.

Multiple architectural and training configurations were tested to evaluate the influence of parameters such as the number of hidden layers, the number of neurons per layer, activation functions, optimization algorithms, and other hyperparameters on predictive performance. Performance was evaluated using two standard deviation metrics: root-mean-squared error (RMSE) and mean absolute error (MAE), defined in eqs 2 and ([Disp-formula eq3]). In addition, parity plots were used to visually assess the quality of the predictions and to identify potential systematic deviations. These metrics were monitored during training and guided the architecture selection.

2 $$RMSE=\sqrt{\frac{1}{n}\sum_{i=1}^{n}{\left(y_{i}-{ŷ}_{i}\right)}^{2}}$$

3 $$MAE=\frac{1}{n}\sum_{i=1}^{n}\left|y_{i}-{ŷ}_{i}\right|$$

Across all tests, networks structured with two processing layers consistently achieved the lowest errors—likely due to the enhanced ability to identify hierarchical nonlinearities in the variables—and were therefore implemented. The number of neurons per layer was explored interactively over a broad range. Initial tests focused on smaller architectures (up to 50 neurons per layer), which quickly reached a performance plateau. Intermediate configurations (up to 100 neurons per layer) showed no further gains. Notably, increasing the number of neurons in this range did not produce a systematic rise in the validation error, as would be expected from overfitting but rather yielded fluctuating results without consistent improvement. Substantial error reduction was observed only with larger architectures (300–500 neurons per layer), which ultimately justified the use of deep learning.

Regarding activation functions, more than 10 alternatives were tested, including widely used options such as ReLU, Leaky ReLU, and SELU. Among them, the Gaussian Error Linear Unit (GELU) was selected for the intermediate layers due to its differentiable, nonmonotonic characteristics, which facilitate improved gradient flow and enhance convergence stability, promoting more effective pattern identification.^18^ At the output layer, the sigmoid function (σ) was used for the output consistency. Both functions are presented in eqs 4 and ([Disp-formula eq5]). Furthermore, model training was performed using the Layerwise Adaptive Moments optimizer for Batch training (LAMB), which demonstrated stable convergence and lower validation error across multiple trials.

4 $$GELU\left(x\right)=\frac{x}{2}\left[1+\mathrm{erf}\left(\frac{x}{\sqrt{2}}\right)\right]$$

5 $$\sigma \left(x\right)=\frac{1}{1+e^{-x}}$$

Finally, to mitigate overfitting—a critical concern given the limited data set size—regularization strategies were employed throughout training. Early stopping algorithms were implemented by monitoring validation loss to halt training once performance plateaued, thereby preserving generalization and avoiding overadaptation to the training data. In addition, although cross-validation techniques were initially tested, they did not improve performance, likely due to noise introduced by repeated random splits in a small data set. As a result, a fixed 80–20 split was adopted for training and validation, respectively. In addition, external validation was conducted using a separate set of experiments not used during training and internal validation, providing an independent assessment of the model’s reliability outside the domain of data originally used in its construction.

### 2.3 Sensitivity Analysis

Sensitivity analysis is a key strategy for evaluating data-driven models, particularly in systems in which underlying physicochemical laws are not explicitly encoded. In the context of neural networks, it enables the assessment of the influence of specific variables on the system output. Essentially, this evaluation consists of introducing systematic perturbations to input variables in order to observe the model response to isolated variations, while other conditions remained predetermined at representative operating values.

In this study, a series of simulations was conducted in which selected input variables were independently perturbed over defined ranges to isolate their individual effects on the predicted ammonia conversion. The perturbed variables included all process-related inputs—reaction temperature, WHSV, and ammonia concentration in the feed stream (In % NH_3_). For each variable, five catalysts with different active phase metals were analyzed to assess their influence and identify the most effective compositions. In addition, the influence of the metallic content in the catalyst active phase was examined through variations in the weight fraction of Ruthenium (Ru). These particular investigations, regarding catalyst compositional effects on the reaction outcome, would be impractical to conduct using conventional theory-based models. Table 2 displays the selected variables and their respective varying ranges.

**Table 2:** Selected Variables and Corresponding Ranges for Sensitivity Analysis Using the Developed Neural Network Describing the Catalytic Decomposition of Ammonia

variable	unit	min value	max value
temperature	°C	300	650
WHSV	mL g^–1^h^–1^	60	100,000
in % NH_3_	vol %	1	100
Wt % active phase (Ru)	wt %	1	10

For reference and posterior comparisons, baseline conditions were established and fixed for the variables that were not being varied in the corresponding sensitivity analysis. For numeric variables, the most frequent values reported across different studies were selected as they were more accurately represented in the data set. As for the catalyst support, aluminum oxide (Al_2_O_3_) was also chosen due to its high representation in the experimental data. Accordingly, Table 3 presents the baseline conditions for all of the variables.

**Table 3:** Baseline Conditions for Sensitivity Analysis Using the Developed Neural Network Describing the Catalytic Decomposition of Ammonia

variable	unit	value/identity
temperature	°C	500
WHSV	mL g^–1^ h^–1^	16,000
in % NH_3_	vol %	100
active phase	–	Ru, Ni, Fe, Co, Mo
Wt % active phase	wt %	10
support	–	Al_2_O_3_
Wt % support	wt %	90

This analysis enabled the generation of parametric response curves that characterize the model’s sensitivity to each input. Beyond confirming the alignment of predictions with established physicochemical trends, the procedure offers insight into nonlinear behavior, response saturation, and feature interactions recognized by the network. It also serves as a diagnostic tool, enabling the assessment of whether the model responds to systematic variations in consistency with physical intuition and literature-based expectations.

## 3 Results and Discussion

### 3.1 Model Evaluation

The performance of the ANN models was evaluated through a structured architecture optimization process that systematically varied the structural and internal parameters, as previously described. In the final selection phase, topologies with different numbers of neurons in the intermediate layers were tested to assess their effect on the prediction accuracy and generalization. The objective was to identify a configuration that minimized error while maintaining stable performance across data sets. Figure 2 summarizes the results, presenting the RMSE and MAE values for each topology. For every configuration, training and validation errors are shown, enabling a direct comparison of performance and consistency.

**Figure 2:**
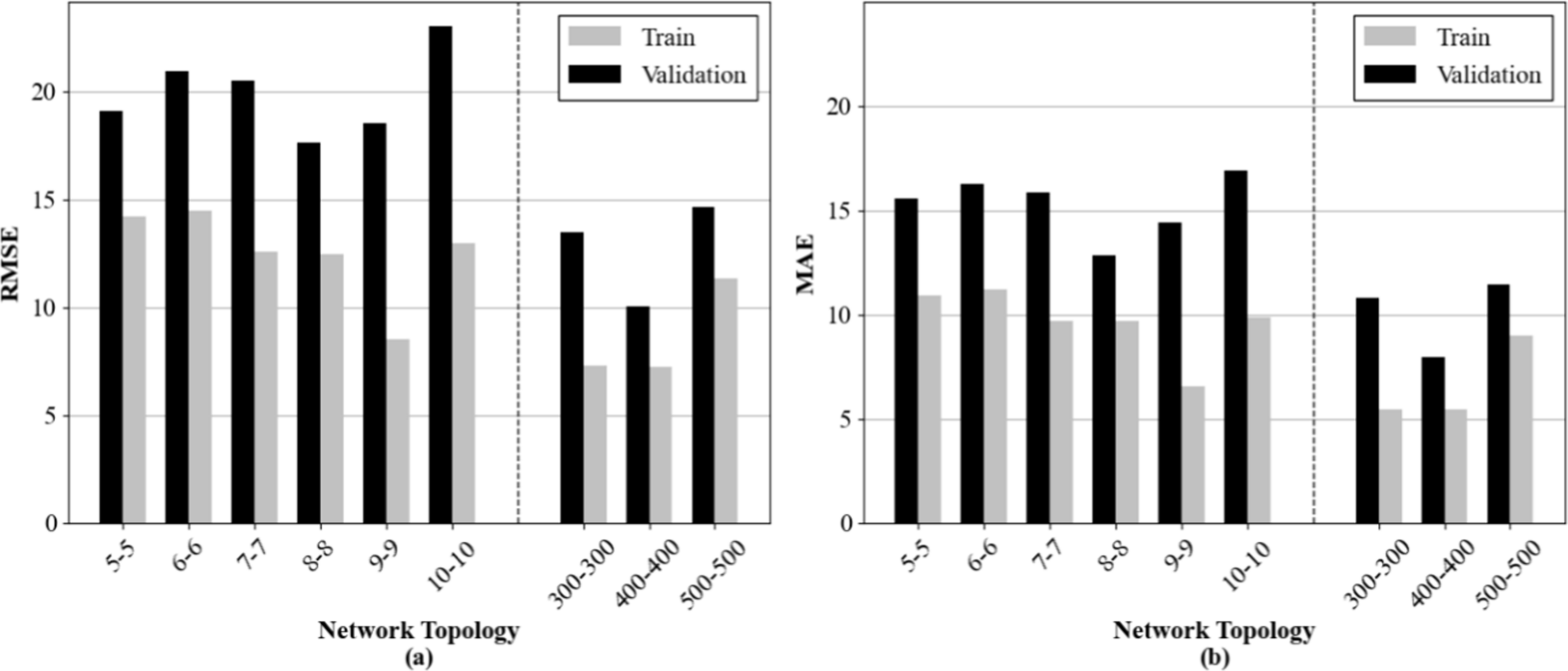
– Evaluation of ANN architectures using (a) RMSE and (b) MAE across different topologies. The notation *x*-*y* labeling the horizontal axes represents the number of neurons in the (*x*) first and (*y*) second intermediate layer.

Overall, simpler architectures—positioned on the left side of the plots—exhibited substantially higher errors, reflecting their limited capacity to capture system complexity. Among these configurations, no consistent performance improvement was observed as the neuron count increased, suggesting oscillations in the balance between the fitting capacity and generalization. In contrast, a clear performance gain was observed with deeper architectures: networks structured with 300, 400, and 500 neurons in each processing layer consistently achieved lower RMSE and MAE values. Notably, these models not only minimized both error metrics but also displayed the smallest difference between training and validation results, indicating that the regularization strategies employed were effective in controlling overfitting while preserving predictive accuracy.

Among all configurations tested, the 400–400 architecture produced the best overall performance. It achieved the lowest validation errors—RMSE = 10.06 p.p. and MAE = 7.98 p.p.—while maintaining a minimal gap between training and validation metrics. This consistency, along with the model reproducibility across multiple runs, supported its selection as the final architecture. Figure 3 presents the corresponding parity plots comparing predicted and experimental ammonia conversion values for both the training and validation sets. The training data showed a strong correlation (*R* ^2^ = 0.932), confirming the model’s ability to capture the system’s underlying complexity. In the validation set, a slightly lower fit was observed (*R* ^2^ = 0.830), with increased dispersion particularly in the intermediate conversion range, likely due to a higher density of overlapping conditions and greater variability in experimental data within this region.

**Figure 3:**
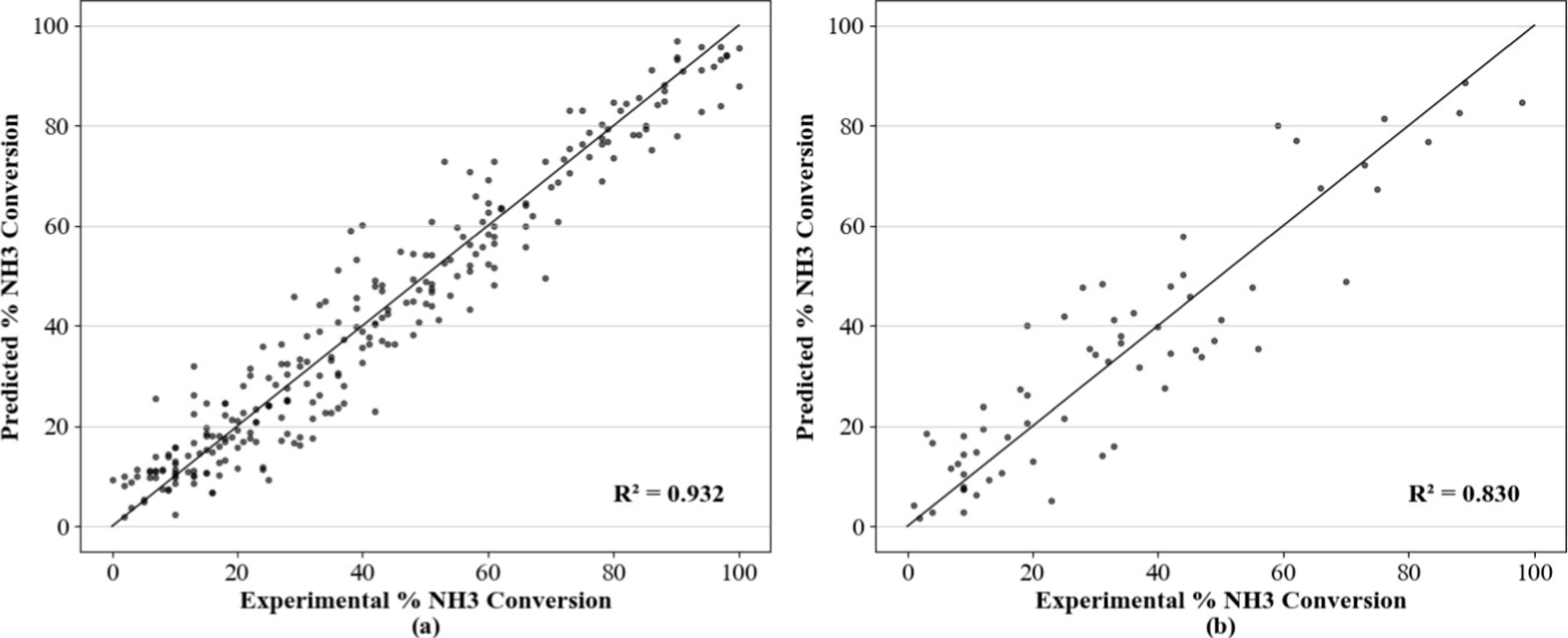
Parity plots for the selected model with 400–400 architecture on (a) training and (b) validation.

While the *R* ^2^ value slightly decreased in the validation data set, the model maintained strong generalization capability, with most deviations occurring in concentrated regions. This behavior is consistent with the architecture’s stable validation performance observed during topology optimization. Furthermore, the small deviation between training and validation metrics underscores the model’s generalization capacity—a key attribute for data-driven modeling of complex chemical systems.

Finally, the metrics indicate a predictive performance comparable to literature benchmarks, including the best-performing GBR-ML model reported by Guo et al.,^17^ as summarized in Table 4. It should be noted, however, that the data sets used in the two studies are not identical, even though they may partially overlap, since both were compiled from the literature. Additionally, while the objectives of both works are similar—the prediction of ammonia conversion under different operating conditions and catalysts—the methodologies for representing catalyst characteristics differ substantially. Guo et al.^17^ described the catalyst using only six descriptors (average pore diameter, pore volume, average crystal size, total metal loading, specific surface area, and crystallinity index). In contrast, the ANN model designed in the present study incorporated the encoding of 20 distinct metals constituting the active phase or acting as promoters and of an additional 27 support materials, as presented in Table 1. This broader representation naturally increased the number of input variables, likely contributing to a higher model complexity.

**Table 4:** Comparison of Performance Metrics between the Proposed Deep Learning Model and Traditional Machine Learning Models Reported in the Literature

variable	ANN^a^	SVR^b^	RFR^c^	GBR^d^
MAE	7.98	13.10	10.58	10.31
RMSE	10.06	17.80	13.39	13.24
R^b^	0.83	0.72	0.84	0.85

a Artificial Neural Network, Present study.

b Support Vector Machine, Guo et al.^17^.

c Random Forest Regression, Guo et al.^17^.

d Gradient Boost Regression, Guo et al.^17^.

Both strategies exhibit inherent advantages and limitations. In principle, any catalyst can be characterized through descriptors, and therefore the method adopted by Guo et al.^17^ can, in theory, predict the performance of catalysts of any composition, provided that the descriptors are determined. However, the experimental determination of the descriptors requires prior synthesis and characterization of the catalyst. Moreover, although such descriptors may represent a reasonable attempt to reduce catalyst features, it is possible that catalysts with distinct chemical compositions but similar descriptors may still display different performances. Conversely, the framework proposed in this work enables predictions for novel, not yet synthesized catalysts, serving as a valuable tool to guide the preparation and testing of compositions identified by the model as being most promising. This feature makes the model particularly well-suited for the design and optimization of new catalysts. Its main limitation, however, is that the composition must remain constrained to the chemical elements and materials included in the model development—i.e., those listed in Table 1. Future efforts should focus on combining the advantages of these two methodologies.

### 3.2 Sensitivity Results

To further explore the behavior of the best-performing model—structured with a 400–400 architecture—an extensive sensitivity analysis was carried out to examine its internal logic and responsiveness to key variables. Simulations were conducted for five catalysts containing 10 wt % of different active metals. Figure 4 shows the resulting NH_3_ conversion curves as a function of the operating temperature. The model faithfully reproduces the expected dynamics of an endothermic catalytic system, with conversion increasing consistently with temperature, in accordance with the reaction’s kinetics and thermodynamics.^19^

**Figure 4:**
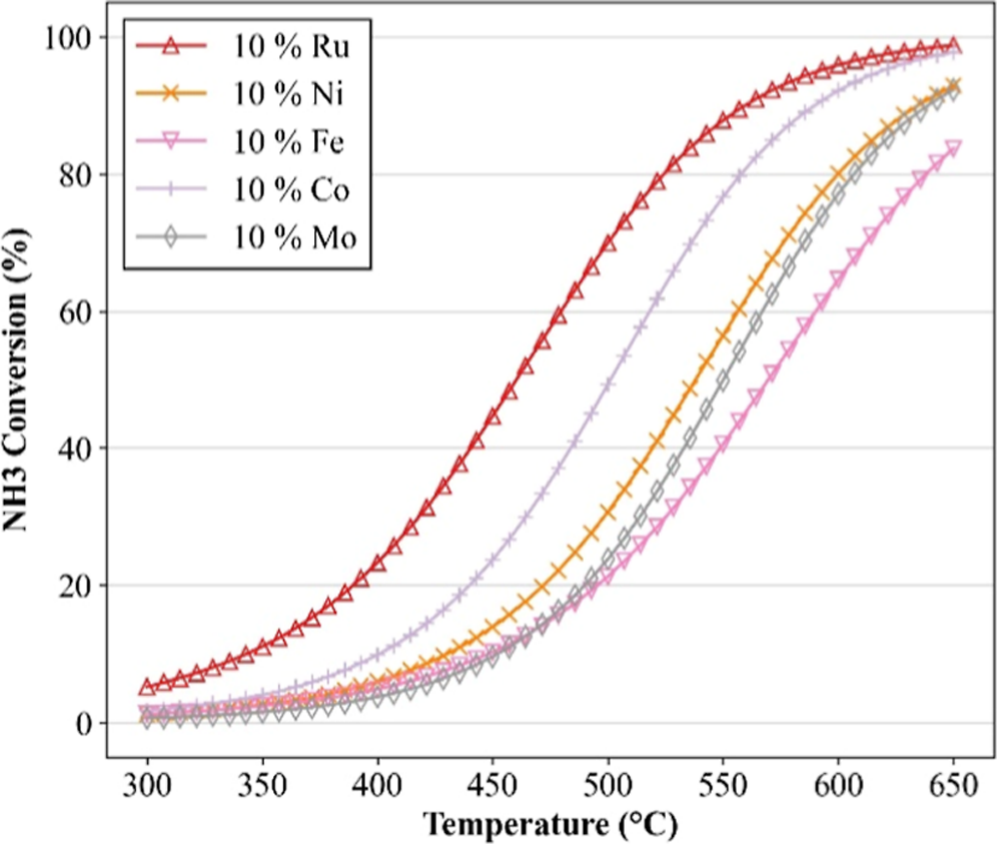
Sensitivity simulations on operating temperature for the catalytic decomposition of ammonia. In all simulations, the following parameters were predetermined: Support of Al_2_O_3_; Wt % Support = 90 (wt %); WHSV = 16,000 (mL g^–1^ h^–1^); In % NH_3_ = 100 (vol %). Markers are used solely for improved visualization.

The clear separation between the conversion curves further highlights the model’s ability to distinguish the influence of the active phase identity, which governs the availability and reactivity of surface sites involved in N–H bond cleavage and N–N recombination. Across all simulations, Ru exhibits the highest conversion over the entire temperature range, confirming its superior performance as the active metal and reinforcing its strongly supported efficiency in promoting ammonia decomposition.^8^ This behavior is consistent with its favorable electronic properties, which lower the activation energy barriers associated with these elementary steps.^20^

The model’s response to changes in WHSV is presented in Figure 5, using the same five catalysts evaluated in the temperature analysis. The simulations exhibit a clear inverse relationship between WHSV and ammonia conversion, with a sharp decline at low WHSVs followed by stabilization at higher flow rates—a trend consistent with the typical kinetics of continuous-flow catalytic systems.^19^ As the WHSV increases, the residence time of reactants in the catalytic bed decreases, reducing the time available for reaction and thereby limiting conversion.

**Figure 5:**
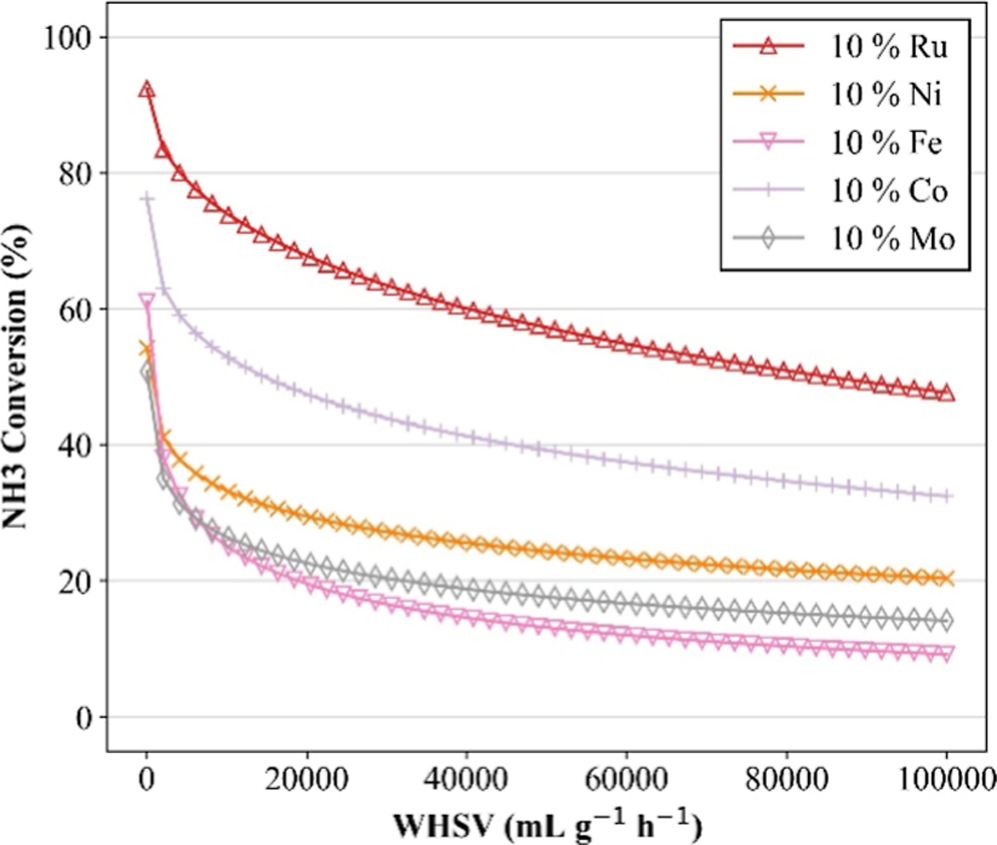
Sensitivity simulations on WHSV for the catalytic decomposition of ammonia. In all simulations, the following parameters were predetermined: support of Al_2_O_3_; Wt % support = 90 (wt %); in % NH_3_ = 100 (vol %); temperature = 500 (°C). Markers are used solely for improved visualization.

Figure 6 illustrates the model response to variations in the ammonia feed concentration—an operational parameter of particular relevance because of its role in thermal management and reaction control via inert gas dilution.^21^ For all of the catalysts analyzed, increasing the NH_3_ fraction led to a gradual decrease in conversion. This pattern aligns with physicochemical expectations: elevated NH_3_ levels promote active site saturation, hindering competitive adsorption and reducing the overall reaction rate—especially in systems where desorption is rate-limiting. The introduction of inert gases, by contrast, aids thermal dissipation and can facilitate desorption kinetics.^21^

**Figure 6:**
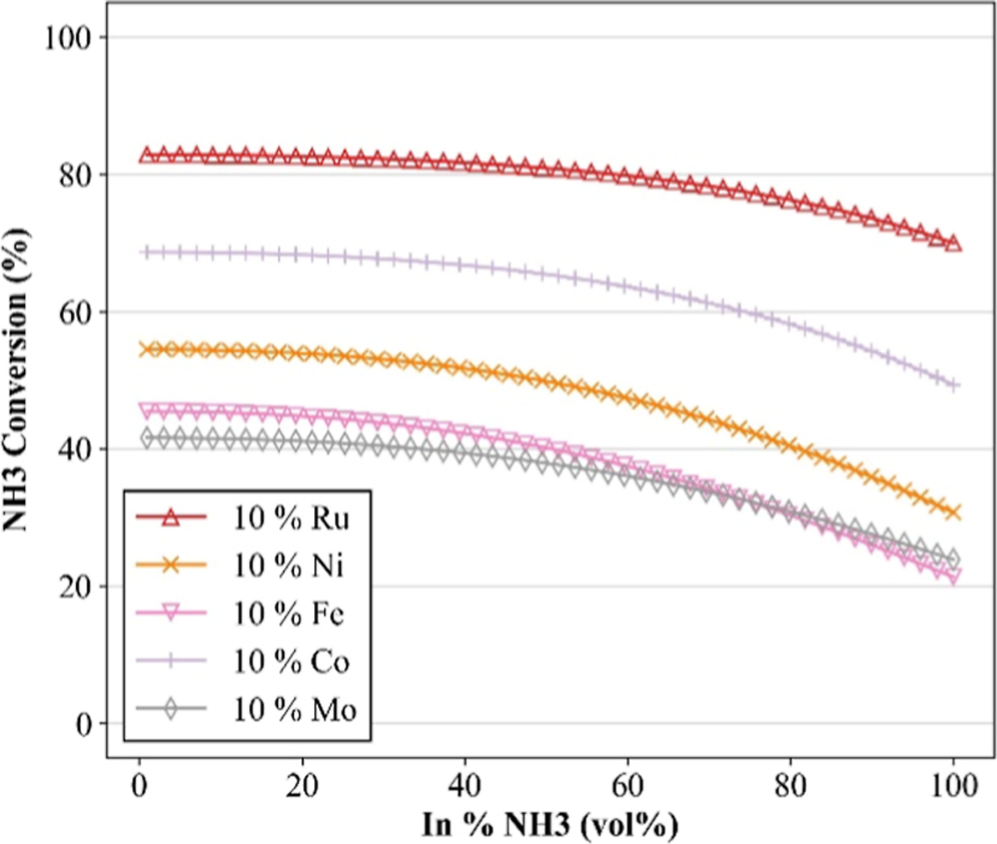
Sensitivity simulations on the ammonia feed concentration for the catalytic decomposition of ammonia. In all simulations, the following parameters were predetermined: support of Al_2_O_3_; Wt % support = 90 (wt %); WHSV = 16,000 (mL g^–1^ h^–1^); temperature = 500 (°C). Markers are used solely for improved visualization.

Figure 7 exhibits the model response to variations in the mass fraction of ruthenium (Ru) in the catalyst active phase. Metal loading was systematically varied from 1 to 10 wt %, and the support fraction was adjusted accordingly from 99 to 90 wt %, while all other structural and operational parameters were defined at representative conditions. Conversion increased steadily with the Ru content, though a clear saturation effect was observed at higher loadings. The trend is consistent with established catalytic principles, in which an increase in active site density enhances reaction rates up to a point beyond which limitations such as internal diffusion, site blockage, or metal agglomeration start to attenuate the benefits of further additions.^19^

**Figure 7:**
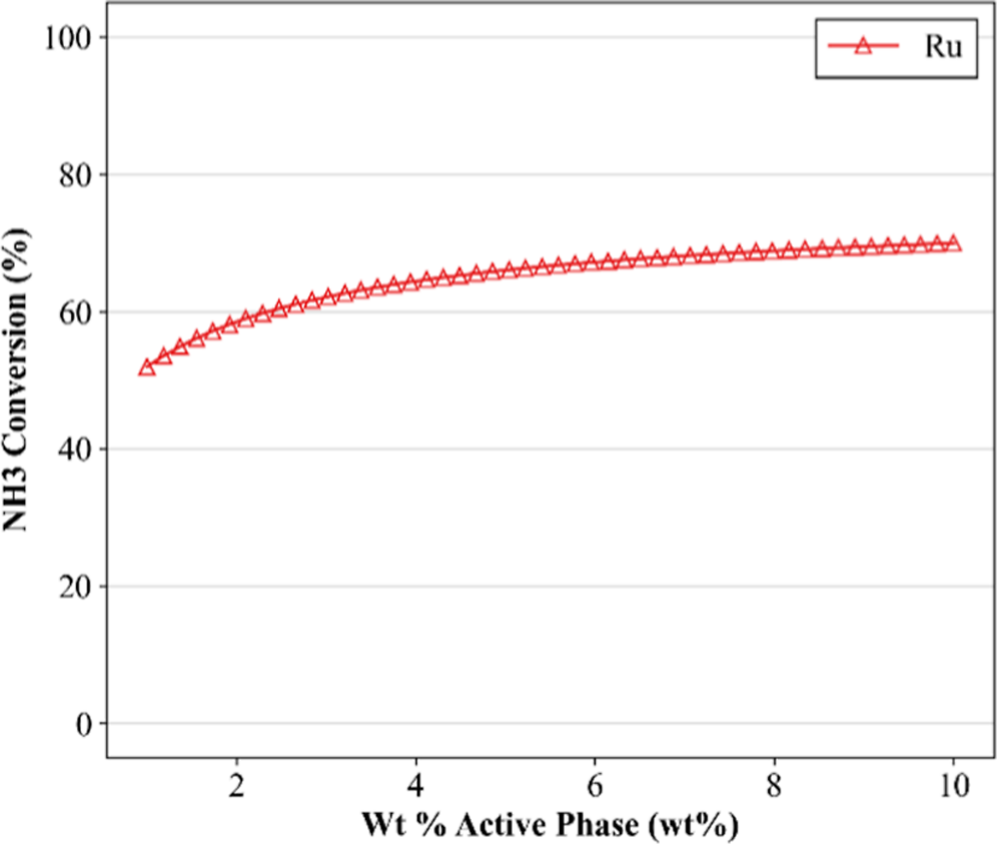
Sensitivity simulation on metal loading in the active phase for the catalytic decomposition of ammonia. In all simulations, the following parameters were predetermined: support of Al_2_O_3_; Wt % support ranged from 99 to 90 (wt %); WHSV = 16,000 (mL g^–1^ h^–1^); in % NH_3_ = 100 (vol %); temperature = 500 (°C). Markers are used solely for improved visualization.

Overall, the sensitivity simulations consistently revealed the model capacity to reproduce key behaviors expected of catalytic ammonia decomposition. Its responses to perturbations in operating variables—such as temperature, WHSV, and NH_3_ concentration—aligned with extensively documented kinetic and thermodynamic principles, while the generated curves remained stable and smooth, reflecting reliable training. The model also demonstrated refined sensitivity to the catalyst composition, accurately identifying metal-specific effects on the catalyst performance including both metal identity and loading.

A final SHAP analysis further substantiates the model interpretability and reflects the trends observed in the sensitivity simulations. Figure 8 presents the SHAP values for all main variables, including the operating parameters—temperature, WHSV, and ammonia feed concentration—and the catalyst composition—active phase, support, and promoters. Fundamentally, SHAP quantifies the contribution of each input to the model output, providing a clear ranking of the feature importance. The analysis was performed on the entire data set, ensuring comprehensive representation of all conditions internalized by the model.

**Figure 8:**
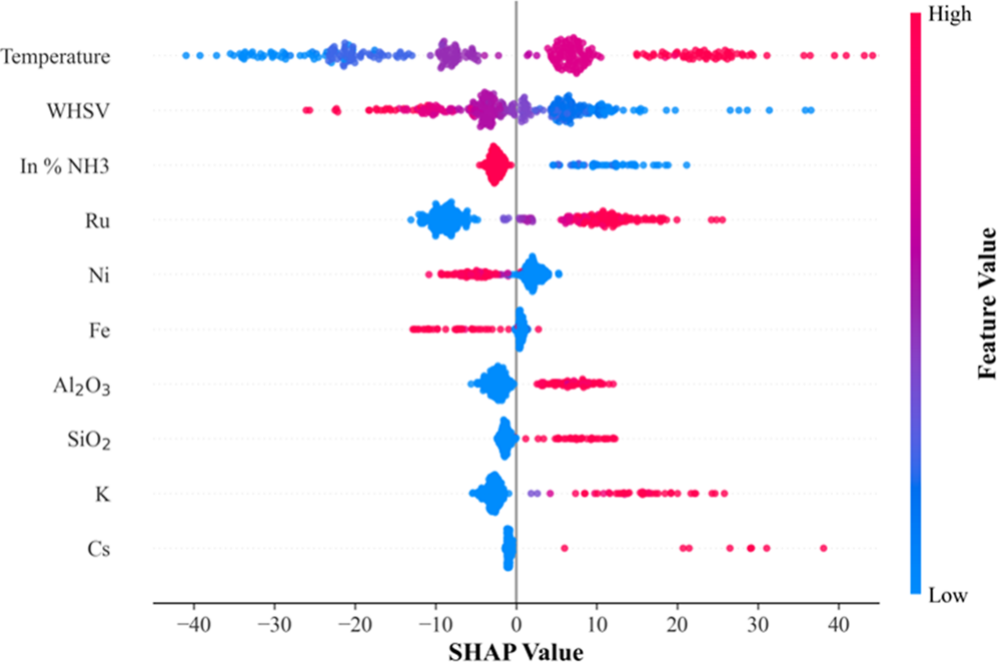
SHAP analysis showing the contributions of the main input variables across four categories: operating variables; active phase identity; support identity; and promoter identity (top to bottom in the figure). Within each category, variables are ranked by the magnitude of SHAP values.

As expected, operating parameters, particularly temperature and WHSV, exhibit the highest impact, confirming their dominant role in ammonia decomposition.^19^ Nonlinear effects are also evident: while higher temperatures enhance conversion, trends for WHSV and ammonia feed concentration reflect diminishing returns at elevated values, consistent with the sensitivity simulations and with established reaction-engineering principles linking residence time and reactant partial pressure to conversion.^19,21^ Compositional features, although generally smaller in magnitude, also influence model predictions: specific metals in the active phase, such as ruthenium, and support oxides, such as Al_2_O_3_, increase conversion at higher concentrations. In addition, the SHAP analysis demonstrates the model’s ability to capture subtle catalyst configurations: promoters such as potassium and cesium have minimal impact when absent but can significantly affect the output when integrated into catalyst formulation.

The results reinforce the model interpretability, consistently demonstrating that its predictions are grounded in real operational principles and supported by experimental observations reported in the literature, highlighting the model’s ability to capture both dominant effects and subtle interactions across operating conditions and catalyst composition.

### 3.3 External Validation

Finally, to evaluate the model’s ability to generalize beyond its construction domain, genuine external validation was performed using experimental data from three independent studies^22–24^ not included in the original data set used in the model design. These studies report ammonia conversion across a range of temperatures for multiple catalysts that differ in both composition and operating conditions. This analysis comprised 18 data points featuring systematic variations in the catalyst composition, designed to probe both the model’s generalization capacity and its sensitivity to subtle perturbations. These variations included different fractions of active-phase metals and the presence of promoter agents. Additionally, the external validation set contained a complex multioxide catalyst, enabling the assessment of the model’s ability to predict the behavior of entirely new components based solely on the encoded elementary oxides. Operationally, the experiments also include different WHSV conditions while maintaining comparable feed concentrations.

Figure 9 presents the comparison between the model predictions and the experimental results.

**Figure 9:**
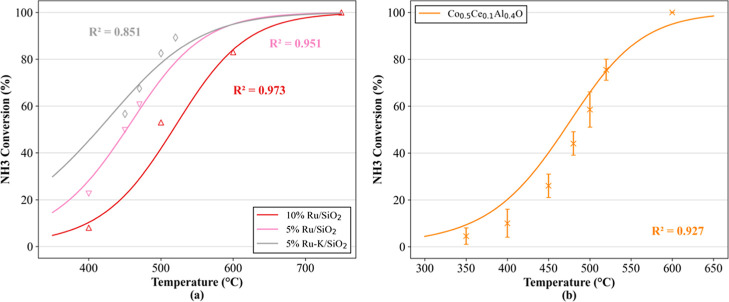
Comparison of model simulations (continuous curves) with external experimental data for the catalytic decomposition of ammonia. Data are not used in the model training. All experiments were conducted with In % NH_3_ = 100 (vol %). (a) Markers represent experimental data (△) from ref 22 with WHSV = 360,000 (mL g^–1^ h^–1^) and (▽, ◊) from ref 23 with WHSV = 30,000 (mL g^–1^ h^–1^). Adapted from ref 22 (Int. J. Hydrogen Energy, 39(2), Varisli, D & Elverisli, E.E.; Synthesizing hydrogen from ammonia over Ru-incorporated SiO_2_-type nanocomposite catalysts, p. 10399-10408), Copyright 2014, with permission from Elsevier. (b) Markers represent experimental data with the respective error bars (×) from ref 24 with WHSV = 12,000 (mL g^–1^ h^–1^). Adapted from ref 24 (Chem. Eng. J., 411, Maleki, H., Fulton, M., & Bertola, V.; Kinetic assessment of H_2_ production from NH_3_ decomposition over the CoCeAlO catalyst in a microreactor: Experiments and CFD modeling, 128595), Copyright 2021, with permission from Elsevier.

The consistent alignment across all simulations confirms the model effectiveness to extrapolate with accuracy, reproducing both global conversion trends and catalyst-specific differences despite variations in formulation and operating regime. Specifically, Figure 8b highlights the effectiveness of the proposed encoding strategy, evidencing the model’s ability to precisely characterize a previously unseen multioxide catalyst based solely on the identities of its constituent oxides.

The external validation analysis further demonstrates the model consistency and reliability, supporting its potential not only as a predictive instrument but also as a resource for interpreting catalytic systems and supporting decision-making in material screening and process optimization.

## 4 Conclusions

This work presents the development of a neural network to model the catalytic decomposition of ammonia, contributing to advances in green hydrogen technologies. The model was trained on a rigorously processed data set comprising 324 experiments, marked by substantial diversity in both catalyst composition and operating conditions. The final data set included over 20 different metals represented in active phases and promoters, along with 27 distinct support compounds, supporting broad coverage of relevant experimental configurations—reflecting the study’s central priority of ensuring wide model applicability.

Based on this data set, a deep learning neural network was developed, consisting of two intermediate layers with 400 neurons each, GELU activation between processing layers, and a sigmoid function at the network’s output, trained using the LAMB optimizer. This architecture was selected by following an extensive comparative analysis of different topologies and hyperparameters. The resulting model achieved solid predictive performance, with an RMSE of 10.06 ppm and an MAE of 7.98 ppm on the validation set. Parity analysis further supported these results, showing a coefficient of determination, *R* ^2^, of 0.932 for training and 0.830 for validation—indicative of reliable generalization. External validation using independent experimental data reinforced the model’s reliability, revealing strong agreement between predicted and observed values across multiple catalytic systems that extended beyond the conditions used in model development.

Sensitivity simulations demonstrated the model’s ability to accurately reproduce well-established physicochemical trends, including the positive effects of increasing temperature and metal loading, the negative influence of higher WHSV and feed ammonia concentration, and the distinct performance of different active metals. This consistent alignment with expected behavior suggests that the neural network not only interpolated the training data but also internalized the fundamental catalytic principles governing the system.

Nevertheless, the use of complex architectures—such as the one employed in this study—raises important considerations regarding interpretability and the suitability of deep learning models for limited data sets. The results obtained, however, suggest that model performance is largely attributable to the quality of data engineering and the rigor of preprocessing rather than to architectural complexity alone. This demonstrates that success in such applications is not exclusively associated with the network’s depth and complexity but with the balanced integration of model design, data quality, and process interpretation.

Future work should focus on expanding and diversifying the data set, particularly by incorporating variables previously excluded due to missing data—such as apparent activation energy, hydrogen production rate, and TOF—as well as additional metals and support materials with insufficient representation. Including these variables could enable multioutput modeling strategies and further enhance the model’s predictive capabilities.

In summary, this study demonstrates that deep learning neural networks provide a powerful framework for modeling complex catalytic reactions. Specifically, these models support the modeling of highly diverse and limited data sets, which often characterize catalytic systems. The developed encoding strategy enables the model to directly incorporate a wide range of catalyst compositions through their constituent oxides or other components. This methodology was consistently effective even for complex multioxide catalyst formulations analyzed in external validation. As a result, the model developed in this work provides valuable strategies for optimizing catalyst screening and design, contributing to the advancement of green hydrogen production and broader energy transition efforts.

## Supplementary Material





## Notes

Workflow for data processing and encoding using representative examples (PDF)
Full final encoded data set for reference and reproducibility (XLSX)
