# Supplementary material for: Deep Learning Kinetic Modeling of the Catalytic Decomposition of Ammonia in Green Hydrogen Production: Effects of Catalyst Composition and Operating Variables
Source: ACS Omega. 2026 Apr 10;11(15):22678–87. doi: 10.1021/acsomega.5c11092 (PMC13103821; doi:10.1021/acsomega.5c11092)
Supplement: Supplementary file 1 [file ao5c11092_si_001.pdf]

Deep Learning Kinetic Modeling of the Catalytic Decomposition of Ammonia in Green Hydrogen Production: Effects of Catalyst Composition and Operating Variables

Felipe Vicent Dalcamim [1], Isabela Belapetravicius [1], Fabio M. Cavalcanti [2], Reinaldo Giudici [1,*]

[1] Universidade de São Paulo, Escola Politécnica, Department of Chemical Engineering, Av. Prof. Luciano Gualberto 380, São Paulo, SP, 05508-010, Brazil

[2] Department of Chemical Engineering, Center of Technology and Geosciences, Institute for Petroleum and Energy Research, Laboratory of Refining and Cleaner Technology, Federal University of Pernambuco, Recife, PE, 50740-540, Brazil

1 Supporting Information

Tables S1–S3 document the complete workflow for data processing and encoding using representative examples. The same data entries are tracked across all tables to enable a step-by-step visualization of the applied transformations.

In addition, an Excel file entitled *"Encoded Database"* is provided as supplementary material. This file contains the full final dataset following the processing and encoding procedures and is included for reference and reproducibility.

Table S1 – Example of part of the original database

Baseline dataset sourced from Lucentini et al. 2019 [8].

Table S1 – Example of part of the original database

Active Phase	Wt % Active Phase	Support	Promoter	WHSV (mL g^{-1} h^{-1})	GHSV (h^{-1})	In % NH_3	Temp. (°C)	Conv. NH_3 (%)	H_2 rate (mmol g^{-1} s^{-1})	E_a (kJ mol^{-1})	TOF (s^{-1})	Ref.
Ni	N.a.[1]	SBA–15	Ce	30000	N.a.	100	500	66	N.a.	N.a.	N.a.	-262
Ni	5.0	SBA–15	K	30000	N.a.	100	500	23	N.a.	N.a.	N.a.	-164
Ni	N.a.	SBA–15	La	30000	N.a.	100	500	59	N.a.	N.a.	N.a.	-262
Ni	5.2	Sepiolite		2000	N.a.	100	550	82	0.03	105	N.a.	-274
Ni	N.a.	SiO2		N.a.	20600	100	450	4	N.a.	N.a.	N.a.	-241
Ni	10.0	SiO2		30000	N.a.	100	450	4	0.02	N.a.	1.0	-158
Ni	10.0	SiO2		6000	N.a.	100	500	31	N.a.	N.a.	N.a.	-256
Ni	10.0	SiO2		36000	1200	100	550	50	N.a.	108	N.a.	-268
Ni	65.0	SiO2–Al2O3		30000	N.a.	100	450	9	0.05	92	N.a.	-158

[1]N.a. = Not available

Table S2 – Example of part of the processed dataset

Chemical formulas and notations were standardized. Rare catalyst compositional features (<3 occurrences, e.g., Sepiolite) were represented by their corresponding elemental oxides. Additionally, the processing phase included the selection of statistically reliable variables and experiments.

Table S2 – Example of part of the processed dataset

Active Phase	Wt % Active Phase	Support	Wt % Support	Promoter	Wt % Promoter	WHSV (mL g^{-1} h^{-1})	In % NH_3	Temp. (°C)	Conv. NH_3 (%)	Ref.
Ni	10.0	SBA15	82.8	Ce	7.2	30000	100	500	66	-262
Ni	5.0	SBA15	88.3	K	6.7	30000	100	500	23	-164
Ni	10.0	SBA15	82.9	La	7.1	30000	100	500	59	-262
Ni	5.2	SiO2-MgO-Al2O3	38.0-13.7-2.0			2000	100	550	82	-274
—[1]	—	—	—	—	—	—	—	—	—	—
Ni	10.0	SiO2	90.0			30000	100	450	4	-158
Ni	10.0	SiO2	90.0			6000	100	500	31	-256
Ni	10.0	SiO2	90.0			36000	100	550	50	-268
Ni	65.0	SiO2-Al2O3	17.5-17.5			30000	100	450	9	-158

[1]Excluded due to missing critical information

Table S3 – Example of part of the encoded dataset

Unique catalyst compositional features are transformed into a input variables representing its weight fraction in the overall catalyst formulation. For example, the first experiment (Ref. 262) employed a catalyst containing 10% Ni, 7.2% Ce, and the remainder SBA15. In the encoded dataset, entries are provided for each present feature (Ni, Ce, SBA15), while all features not present in the catalyst are assigned a value of 0.

Table S3 – Example of part of the encoded dataset

Ag	•••	Ce	•••	K	La	•••	Ni	•••	Al_2O_3	•••	MgO	•••	SBA15	SiO_2	•••	WHSV (mL g^{-1} h^{-1})	In % NH_3	Temp. (˚C)	Conv. NH_3 (%)	Ref.
0.0	•••	7.2	•••	0.0	0.0	•••	10.0	•••	0.0	•••	0.0	•••	82.8	0.0	•••	30000	100	500	66	-262
0.0	•••	0.0	•••	6.7	0.0	•••	5.0	•••	0.0	•••	0.0	•••	88.3	0.0	•••	30000	100	500	23	-164
0.0	•••	0.0	•••	0.0	7.1	•••	10.0	•••	0.0	•••	0.0	•••	82.9	0.0	•••	30000	100	500	59	-262
0.0	•••	0.0	•••	0.0	0.0	•••	5.2	•••	2.0	•••	13.7	•••	0.0	38.0	•••	2000	100	550	82	-274
—[1]	—	—	—	—	—	—	—	—	—	—	—	—	—	—	—	—	—	—	—	—
0.0	•••	0.0	•••	0.0	0.0	•••	10.0	•••	0.0	•••	0.0	•••	0.0	90.0	•••	30000	100	450	4	-158
0.0	•••	0.0	•••	0.0	0.0	•••	10.0	•••	0.0	•••	0.0	•••	0.0	90.0	•••	6000	100	500	31	-256
0.0	•••	0.0	•••	0.0	0.0	•••	10.0	•••	0.0	•••	0.0	•••	0.0	90.0	•••	36000	100	550	50	-268
0.0	•••	0.0	•••	0.0	0.0	•••	65.0	•••	17.5	•••	0.0	•••	0.0	17.5	•••	30000	100	450	9	-158

[1]Excluded due to missing critical information
